# Novel Aspects Concerning the Functional Cross-Talk between the Insulin/IGF-I System and Estrogen Signaling in Cancer Cells

**DOI:** 10.3389/fendo.2015.00030

**Published:** 2015-03-06

**Authors:** Paola De Marco, Francesca Cirillo, Adele Vivacqua, Roberta Malaguarnera, Antonino Belfiore, Marcello Maggiolini

**Affiliations:** ^1^Department of Pharmacy, Health and Nutritional Sciences, University of Calabria, Rende, Italy; ^2^Endocrinology, Department of Health, University Magna Graecia of Catanzaro, Catanzaro, Italy

**Keywords:** insulin/IGF system, GPR30/GPER, estrogen receptor, cancer cells, cancer-associated fibroblasts, signal transduction

## Abstract

The insulin/IGF system plays an important role in cancer progression. Accordingly, elevated levels of circulating insulin have been associated with an increased cancer risk as well as with aggressive and metastatic cancer phenotypes. Numerous studies have documented that estrogens cooperate with the insulin/IGF system in multiple pathophysiological conditions. The biological responses to estrogens are mainly mediated by the estrogen receptors (ER)α and ERβ, which act as transcription factors; however, several studies have recently demonstrated that a member of the G protein-coupled receptors, named GPR30/G-protein estrogen receptor (GPER), is also involved in the estrogen signaling in normal and malignant cells as well as in cancer-associated fibroblasts (CAFs). In this regard, novel mechanisms linking the action of estrogens through GPER with the insulin/IGF system have been recently demonstrated. This review recapitulates the relevant aspects of this functional cross-talk between the insulin/IGF and the estrogenic GPER transduction pathways, which occurs in various cell types and may account for cancer progression.

## Physiology of the Insulin/IGF System

IGF-1 and IGF-2 (IGFs) as well as insulin and cognate receptors (the type I IGF-1 receptor, IGF-1R, and the insulin receptor, IR) belong to a complex system, the insulin/IGF system, which is essential for normal development and growth of cells, organs, and whole animals (1). Insulin, which secretion is modulated by nutrients, namely glucose, is exclusively produced by the endocrine pancreas and is the main regulator of glucose homeostasis. The insulin pro-hormone, proinsulin, is partially also secreted in the bloodstream and its biological role, if any, is unknown (2). IGF-1, which is regulated by the hypothalamus/pituitary axis through pituitary GH, is mainly produced by the liver and plays a major role in linear growth. IGF-2 is produced in the liver and many other tissues, and is scarcely responsive to GH. It is mainly involved in trophic, survival, and differentiation effects (3). IGFs, but not insulin or proinsulin, associate with six different binding proteins (IGFBPs) that regulate their bioactivity. IGFs act on most cells and tissues and regulate survival, mitogenesis, cell migration, and differentiation (3).

Insulin and IGFs all activate a very well-conserved signaling pathway, the PI3K/Akt/FoxO pathway, which has a crucial role in the regulation of metabolism, growth, and apoptosis processes (4). In particular, upon ligand binding, the tyrosine kinase domains of both IR and IGF-1R catalyze the phosphorylation of specific substrates, such as the insulin receptor substrate (IRS)-1, IRS-2, IRS-3, and IRS-4, Gab-1, Cbl, and Shc (5). Activated IRS proteins interact with the growth factor receptor binding protein 2 (GRB2) and the p85 regulatory subunit of phosphoinositide 3-kinase (PI3K), which then activates the Akt pathway that regulates metabolic enzymes and mediates cell growth, proliferation, and survival (4). Moreover, IR and IGF-1R activate a second major signaling pathway, the Ras/Raf/MEK/ERK transduction cascade, which is involved in important biological responses like gene expression, cell motility, proliferation, survival, differentiation, and death (6). Another pathway, which integrates signals from both the PI3K–Akt activation and the Ras/Raf/MEK/ERK, as well as signals coming from other growth factors and from nutrients, is the mammalian target of rapamycin (mTOR) pathway, which is also a central regulator of cell growth and metabolism through the control of mRNA translation (7). Moreover, insulin/IGFs also cooperate with other receptor and signaling pathways, thus exerting a crucial role in normal development and homeostasis and tissue repair.

## Involvement of the Insulin/IGF System in Cancer

It is now well established that the insulin/IGF system is frequently dysregulated in cancer, thus contributing to cancer progression, metastases, and resistance to cancer therapies (8, 9). Common alterations include overexpression of IR and IGF-1R by the malignant cells, increased IR/IGF-1R hybrid formation, deregulated autocrine secretion of IGFs, and increased IGFs secretion by the tumor stroma. IGFBPs production in the tumor microenvironment may also be dysregulated (8, 10–12). Indeed, an overexpression of IR/IGF-1R has been detected in breast malignancy (13, 14), and a high expression of these receptors was shown to be tumorigenic in mouse tumor models (15). Moreover, epidemiological studies have shown that elevated IGF-1 plasma concentrations are associated with a higher risk of developing various malignancies, like breast, colon, prostate, and lung carcinomas (16–18). Notably, the human IR exists in two isoforms characterized by the inclusion (IR-B) or the exclusion (IR-A) of exon 11, which encodes a 12 amino acid residue located at the carboxyl terminus of the IR alpha-subunit (5). In adult life, the metabolic effects of insulin in target organs (liver, muscle, and fat) are predominantly mediated by the IR-B. In contrast, IR-A plays a role in growth and development in prenatal life, while its role in adult life is unknown. Recently, IR-A has been shown to be almost invariably overexpressed in both epithelial and non-epithelial malignancies (19–21). Interestingly, while IR-B is a highly specific receptor for insulin, IR-A is a more promiscuous receptor, exhibiting high affinity for insulin, intermediate affinity for IGF-2 and proinsulin, and low affinity for IGF-1 (19, 22, 23). IR-A overexpression and enhanced IGF-1/IGF-2 autocrine/paracrine production often coexist in several malignancies and is associated with an aggressive and dedifferentiated tumor phenotype (20, 24, 25). In this scenario, it is most likely, that IR-A functions as the main IGF-2 receptor, as the IGF-1R is often saturated by the high levels of IGF-1 of tumor microenvironment. Further corroborating these findings, phosphorylated IR was found in different breast-cancer subtypes and correlated with a poor survival (26). Noteworthy, IGF-1R and IR function as transcriptional regulators of the IGF-1R promoter activity in cells with reduced estrogen receptor (ER) levels (27). In particular, in ER depleted C4.12.5 breast-cancer cells but not in ER-positive MCF-7 cells, IGF-1R and IR were able to bind to the promoter of IGF-1R after translocation into the nucleus. IGF-1R enhanced the activity of its own promoter, whereas IR acted as a negative regulator of the IGF-1R promoter activity (27).

IR-A overexpression in cancer has helped us in explaining recent findings indicating that high levels of circulating insulin (hyperinsulinemia) may be related to cancer. Indeed, various lines of evidence indicate that compensatory hyperinsulinemia associated with insulin resistance is significantly associated with increased risk for various cancer histotypes (28) and are also associated to poor cancer prognosis (29). The “Women’s Health Initiative Study” showed that women exhibiting insulin levels in the upper tertile were more than twice as likely to develop breast cancer compared with those presenting insulin levels in the lowest tertile (30). It has been also shown that increased insulin levels are associated with an augmented risk for benign proliferative breast disease (BPBD) (31). Likewise, the relationship between hyperinsulinemia and breast cancer was demonstrated using an insulin-resistant and hyperinsulinemic transgenic mouse model (32). Insulin resistance and hyperinsulinemia are common in metabolic disorders, such obesity and type 2 diabetes mellitus (T2DM), which are both associated with up to two- to threefold increased risk for various malignancies (33–35). The rising occurrence of obesity and T2DM worldwide is causally associated with changing diet and lifestyle, which cause excessive adiposity, especially in the visceral region. Interestingly, in insulin-resistant states, the increased levels of circulating insulin produce biased biological effects whereby glucose metabolism is impaired whereas proliferative effects are unimpaired or enhanced (36, 37). In fact, obesity and T2DM should be considered low-grade inflammatory disorders, owing to macrophage infiltration of hypoxic visceral adipose tissue, and it is well known that inflammatory cytokines impair the IRS/PI3K/Akt pathway (so called metabolic pathway) but do not affect the Ras/Raf/MEK/ERK pathway, which may produce unbalanced stimulation of cancer cells (38, 39). The most recent World Health Organization (WHO) World Cancer report (2014) acknowledge these findings and states that the association of waist size and BMI with cancer risk follows a dose–response relationship, and that overall cancer mortality also increases in a linear fashion with increasing BMI. There is also evidence for a direct association between T2DM and cancer mortality (35, 38). Therefore, insulin resistance, obesity, and T2DM should be considered major preventable cancer risk factors (40, 41). This increased awareness may have important implications in T2DM patient’s management. Diabetic patients are exposed to endogenous hyperinsulinemia both in the pre-diabetic state and at the early stages of the disease, but also when treated with insulin or insulin analogs at later stages of the disease. Moreover, there is concern that insulin analogs may increase cancer because of a biased effect on IR signaling pathways (42). Because of the recognized role of the insulin/IGF system in cancer, in the last decade, this system has been seriously considered as a therapeutic target (43, 44). Indeed, several blocking strategies have been developed in order to selectively target the IGF-1R, with the aim to block protumoral effects without causing deterioration of glucose metabolism. Although pre-clinical studies and some phase I and II clinical trials have been very promising (45), results from phase III trials were disappointing showing clinical benefits only in small subsets of patients (44). A number of factors account for these disappointing results (46). Major factors include insulin resistance and compensatory hyperinsulinemia and increased IR-A in malignant cells (47). The cross-talk of IR/IGF-1R with matrix receptors and with other signaling pathways involved in induction and maintenance of cell stemness features may also have a role (48). As we will summarize below, previously unappreciated modalities of cross-talk with estrogen transduction pathways may also partially account for resistance to selective IGF-1R inhibitors.

## Cross-Talk between Insulin/IGF System and Estrogens

The insulin/IGF system and estrogens act synergistically as potent mitogens in normal breast as well as in breast tumor cells (49). Originally, it was considered that these agents display their actions through separate pathways, but a growing body of evidence has suggested that the insulin/IGF system and estrogen-mediated signaling pathways are strictly interconnected (49). Both classical and non-classical ERs have been shown to concur to this extensive cross-talk.

### Cross-talk involving classical ERs

Classical ERs include two subtypes, ERα and ERβ, which belong to the nuclear receptor family of transcription factors. Both receptor subtypes exert a role in cancer as suggested by the observation that ERα is overexpressed in breast-cancer cells while ERβ in prostate cancer metastases (50, 51). Both ERα and ERβ may act through ligand-dependent and ligand-independent mechanisms. The first pathway includes the well-known genomic actions and the membrane-initiated rapid effects. The ligand-independent pathway comprises the activation of other signaling effectors, like growth factors, which, after binding to kinase receptors, induce ERs phosphorylation and, thereby, activate them to dimerize, bind DNA, and regulate genes (52). The cross-talk between classical ERs and the IGF system may occur through both ligand-dependent and -independent activation. However, the ligand-independent signaling are the mechanisms especially evident in cancer, where they may contribute to tumor progression and endocrine resistance (53).

Early studies describing a cross-talk between classical ERs and the IGFs pathway were conducted in breast and prostate tumors but may be recapitulated in all tumors where both signaling are simultaneously active in inducing a positive feedback cycle of cell survival and proliferation stimuli. For example, a role of ERα in mediating insulin and IGF-I growth effects, also in absence of estrogens, was found in a pituitary tumor cell line (54) as well as in SK-ER3 neuroblastoma cells (55). In human breast-cancer cells, ligand-dependent stimulation of ERs has been shown to enhance IGF signaling at multiple levels (56). For instance, 17β-Estradiol (E_2_) upregulates the expression of several IGF family members including IGF-1, IGF-2, IGF-BP2, IGF-1R, and IRS-1, whereas the expression of IGFBP3 decreases upon estrogen exposure (57–61). In addition, activated ERα by E_2_ induces the phosphorylation of IGF-1R, which triggers downstream transduction pathways (62). IRS-1 upregulation by E2 was associated with a direct positive regulatory role on the IRS-1 promoter (60), while IGF-IR upregulation by E2 appears to involve, at least in part, the transcription factor Sp1 (57). In turn, IGF-1 stimulation may induce ligand-independent ER activation by inducing ER phosphorylation. Akt activation appears to be required and a constitutively active Akt was able to mimic IGF-1 effects (63, 64). Other studies indicated that the main molecular mechanism responsible is the activation of the PI3K/mTOR/S6K1 pathway, which phosphorylates ERα at S167 in a mitogen-activated protein kinase (MAPK)-independent manner (65). Phosphorylated ERα^S167^ may bind and stimulate ERE sequences, and promote gene transcription, growth, and proliferation (65). Interestingly, this response was abrogated by the mTOR1 inhibitor rapamycin (65). However, it has also been shown that E2 and IGF-1 differentially regulates ER-dependent transcription both at ERE and AP-1 sites, indicating that the effects of ligand-dependent and ligand-independent ER activation are not identical (66). At least some of these functional interactions between ERs and the IGF system may be recapitulated in other tissues and tumors (67, 68). To reinforce the relevance of the ER–IGF-1 cross-talk in cancer, microarray data have suggested that a gene signature co-regulated by IGF-1 and estrogens associates with poor prognosis in breast cancer, indicating that the inhibition of both IGF-1R and ER may be necessary in certain subtypes of breast cancer (69). Nonetheless, tamoxifen-resistant (TamR) breast-cancer cells may exhibit reduced levels of IGF-1R (70). Thus, in breast malignancies characterized by a tamoxifen resistance, IGF-1R has been proposed as a poor therapeutic target (70).

Another intriguing cross-talk with the IGF system is elicited by the small fraction of classical ER located at the level of the cell membrane and acting via MISS (membrane-initiated steroid signaling). We have recently described a novel mechanism of cross-talk between estrogens and IGF system in prostate cancer cells, involving the upregulation of the IGF-1R through the classical ERs acting via MISS (71). Both ER isoforms behave similarly in activating this pathway that requires the activation of Src, ERK, and PI3K, and results in the phosphorylation of CREB transcription factor. These findings are in close agreement with previous studies indicating that E2 activates a Src-dependent pathway by inducing an interaction between the ER phosphotyrosine 537 and the SH2 domain of Src (72, 73). These authors have also shown that ER, Src, and p85 form a ternary complex, whose assembly is stimulated by E2 (72). In turn, this complex activates both the Src and the PI3K/Akt pathways and will eventually affect gene expression by affecting multiple transcription factors, including Elk-1, c-fos, and down-regulation of C/EBPbeta and c-Jun (74). We found that CREB responsive elements are present in the 5′UTR region of IGF-IR promoter. IGF-1R upregulation by this mechanism is able to enhance IGFs effects in prostate cancer cells (71). Moreover, IGF-IR itself may phosphorylate CREB and induce CREB-dependent genes (75, 76), therefore regulating its own gene expression. Notably, this pathway is only partially blocked by classical anti-androgens or anti-estrogens, which preferentially block the genomic pathway, but it may be sensitive to inhibitors of the Scr/ERK/PI3K/CREB pathway (71) and to the antidiabetic drug metformin, which blocks MISS at multiple levels (48).

### Cross-talk through the non-classical estrogen receptor GPER

#### GPER action

The G-protein estrogen receptor (GPER), formerly known as G protein receptor 30 (GPR30), mediates rapid responses to estrogens in several types of normal and tumor cells as well as cancer-associated fibroblasts (CAFs) (77–80). Ligand-activated GPER leads to EGFR transactivation and rapid phosphorylation of MAPKs ERK1/2 as well as phosphatidylinositol 3-kinase (PI3K) (81, 82). In addition, GPER signaling stimulates adenylyl cyclase, PKA and PKC activation (83), cAMP accumulation (81, 84), and calcium mobilization (85, 86). The identification of GPER-selective ligands has allowed a better evaluation of GPER-mediated signaling and has further supported the association of GPER with biological responses like gene expression changes, proliferation, migration, and invasion (87–92). In this regard, it has been demonstrated that GPER agonists upregulate the expression of genes associated with tumor progression like c-fos (93–96), cyclins A, D1, and E (97, 98), the connective tissue growth factor (CTGF) (99), and the early growth response-1 (Egr-1) (100). Additionally, GPER has been shown to contribute to the HIF1α-dependent expression of VEGF, which mainly supports angiogenesis and tumor progression (101). It has been also suggested that the localization of GPER at the nuclear level in CAFs occurs via an importin-dependent mechanism and is involved in its transcriptional activity (79, 102). In addition, the potential of GPER in mediating the production of E_2_ in breast CAFs has been recently highlighted (103) together with the observation that hydroxy tamoxifen induces through GPER the aromatase expression in both the SKBR3 breast-cancer cells and CAFs (104). GPER has been implicated not only in cancer but also in cardiovascular, immunological, and neurological functions as well as diabetes (105–107). Accordingly, GPER has been detected in pancreatic β-cells and GPER-ligands have shown insulinotropic effects by mediating pancreatic β-cell survival and stimulating insulin release (108, 109). Pharmacological manipulations and gene deletion of GPER in mice (GPR30^−^/^−^) have been associated with altered insulin release upon estrogen exposure (106, 108). Likewise, GPER deficiency resulted in insulin resistance, dyslipidemia, obesity, and increased circulating pro-inflammatory cytokines, suggesting a role of GPER in metabolism and inflammatory state (110). With regard to the clinical effects mediated by GPER, previous views might be reassessed regarding, for instance, the action of raloxifene and Fulvestrant or Faslodex. Instead of acting solely as ER-modulating agents, these drugs have the potential to act also as agonists for GPER *in vitro* and *in vivo* (111). In this regard, the fact that the activation of GPER causes vasodilation may be consistent with the hypotensive side effects observed in some patients receiving Faslodex (112). Anyway, a better understanding of the Faslodex action is challenging as this compound may act as an agonist of mutated ERα in the activation function-2 (AF-2) (113, 114).

#### GPER regulation and function by the insulin/IGF system

**Figure 1:**
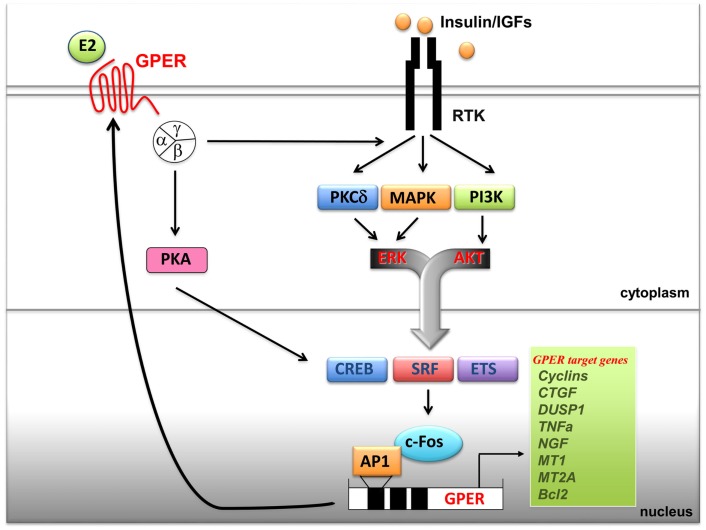
**Cross-talk between GPER and the IGF system**. Upon binding to their specific tyrosine kinase receptors (RTKs), insulin and IGF-I stimulate rapid signals converging on the activation of PI3K, MAPK and PKCδ networks. These pathways, in turn, trigger the activation of transcription factors including CREB, SRF and ETS, which favor c-fos induction and its recruitment to the AP-1 site located next to the GPER 5’ flanking region. Transactivation of GPER promoter sequences induces GPER upregulation at both mRNA and protein levels and, as a consequence, enhanced transcription of GPER target genes. In turn, GPER, upon estrogens binding, activates heterotrimeric G proteins, which trigger multiple effectors including PKA, and also PKCδ, MAPK and PI3K, converging on c-fos induction and GPER gene activation. The resulting effects of these signaling and transcriptional events lead to enhanced mitogenic signals. Abbreviations: PKA, protein kinase A; PKCδ, protein kinase C, δ isoform; MAPK, mitogen activated protein kinases; PI3K, phosphatidyl-inositol-3-kinases; ERK, extracellular signal-regulated kinases; AKT, protein kinase B; CREB, cAMP-response element-binding protein; ETS, E26 transformation specific; SRF, serum response factor; c-fos, FBJ murine osteosarcoma virus; AP-1, activator protein-1; CTGF, connective tissue growth factor; DUSP1, dual specificity protein phosphatase 1; TNFα, tumor necrosis factor α; NGF, nerve growth factor; MT1, metallothionein 1; MT2A, metallothionein 2A; Bcl2, B-cell lymphoma 2.

It has been previously shown that GPER is regulated by EGF and TGFα as well as by hypoxia, one of the main factors involved in tumor aggressiveness (115, 116). Notably, an elevated expression of GPER has been associated with a high risk of metastatic diseases and poor survival rates in breast, endometrial, and ovarian tumors (112). Increased levels of GPER have been also identified in inflammatory breast cancer (IBC), an aggressive hormone-independent form of this malignancy (117). Recently, the overexpression of GPER and its plasma membrane localization were shown to be critical events in breast-cancer progression, whereas the lack of GPER in the plasma membrane was associated with an excellent long-term prognosis in ER-positive tamoxifen-treated breast tumors (118). Therefore, the expression of GPER may characterize not only the estrogen sensitivity and the response to endocrine pharmacological intervention in the above-mentioned tumors but could also be predictive of biologically aggressive phenotypes consistent with adverse outcomes and low survival rates. A cross-talk between the insulin/IGF system and the G protein-coupled receptors (GPCRs) plays a critical role in the regulation of multiple physiological functions and a variety of pathophysiological processes like cardiovascular and renal diseases, obesity, metabolic syndrome, and type II diabetes (78, 112, 119). At the cellular level, insulin as well as IGF-1 dramatically synergizes with GPCR agonists in inducing mitogenic signaling in multiple solid tumors including pancreas, colon, prostate, and breast tumors (119). In addition, recent findings have identified a new cross-talk between the insulin/IGF-1 system and GPER signaling. In particular, IGF-1 has been shown to transactivate the promoter sequence of GPER and to upregulate the expression of GPER at both the mRNA and protein levels in ERα-positive breast (MCF-7) and endometrial (Ishikawa) cancer cells (120). The aforementioned stimulatory action was exhibited by insulin in leiomyosarcoma SKUT-1 cells and in breast CAFs (80). The induction of GPER by both insulin and IGF-1 was mediated by the rapid activation of PKCδ and ERK1/2 transduction pathways and the stimulation of c-fos, which was recruited to the AP-1 site located within the promoter sequence of GPER (Figure 1). The functional role exerted by AP-1 was demonstrated to be essential for the transactivation of the GPER promoter sequence and the GPER upregulation, as the transfection of a construct encoding a dominant-negative form of c-fos abrogated these responses in cell models used. Noteworthy, GPER and one of its main target genes, named CTGF, were required for cell migration induced by IGF-1 and insulin (80, 121). Previous studies have indicated that estrogens increase insulin sensitivity and stimulate glucose uptake in target tissues and breast-cancer cells (122, 123). Of note, GPER has been involved in insulin-regulated metabolic functions in mice and humans (106, 110) as well as in the glucose uptake induced by estrogens (80). In this regard, the insulin-induced expression of GPER was found to boost the glucose uptake stimulated by estrogens and cell-cycle progression (80).

## Conclusion and Perspectives

Many tumors are characterized not only by profound dysregulation of the insulin/IGF axis involving overexpression of receptors, ligands, and intracellular mediators, but also by deregulated expression and trafficking of classical and non-classical ERs and related adaptors/mediators. These conditions greatly enhance the complexity of the cross-talk between the insulin/IGF system and estrogens, which has been largely reported. In this respect, the upregulation of GPER triggered by ligand-activated IGF-1R and IR further contributes to the potentiation of the biological effects induced by estrogens and the insulin/IGF system in cancer. A better understanding of the mechanisms involved in the cooperation of these signaling pathways would provide further opportunities toward innovative anticancer treatments.

## Conflict of Interest Statement

The authors declare that the research was conducted in the absence of any commercial or financial relationships that could be construed as a potential conflict of interest.
